# Supplementary material for: Multifunctional PLA/Gelatin Bionanocomposites for Tailored Drug Delivery Systems
Source: Pharmaceutics. 2022 May 27;14(6):1138. doi: 10.3390/pharmaceutics14061138 (PMC9227928; doi:10.3390/pharmaceutics14061138)
Supplement: Supplementary file 1 [file pharmaceutics-14-01138-s001.zip › pharmaceutics-1652667-supplementary.pdf]

Article

Multifunctional PLA/Gelatin Bionanocomposites for Tailored Drug Delivery Systems

Carmen Moya-Lopez [1], Alberto Juan [2], Murillo Donizeti [1], Jesus Valcarcel [3], José A. Vazquez [3], Eduardo Solano [4], David Chapron [1], Patrice Bourson [1], Ivan Bravo [2], Carlos Alonso-Moreno [2,5], Pilar Clemente-Casares [5,6], Carlos Gracia-Fernández [7], Alessandro Longo [8,9], Georges Salloum-Abou-Jaoude [10], Alberto Ocaña [11,12], Manuel M. Piñeiro [13], Carolina Hermida-Merino [13,*] and Daniel Hermida-Merino [1,13,14,*]

1 Laboratoire Matériaux Optiques Photonique et Systèmes (LMOPS), Centrale Supélec, Université de Lorraine, 57000 Metz, France; carmen.moya-lopez-pelaez@univ-lorraine.fr (C.M.-L.); murillo.donizeti@ufabc.edu.br (M.D.); david.chapron@univ-lorraine.fr (D.C.); patrice.bourson@univ-lorraine.fr (P.B.)
2 Centro Regional de Investigaciones Biomédicas, Unidad NanoCRIB, 02008 Albacete, Spain; alberto.juan@uclm.es (A.J.); ivan.bravo@uclm.es (I.B.); carlos.amoreno@uclm.es (C.A.-M.)
3 Group of Recycling and Valorization of Waste Materials (REVAL), Marine Research Institute (IIM-CSIC), 36208 Vigo, Spain; jvalcarcel@iim.csic.es (J.V.); jvazquez@iim.csic.es (J.A.V.)
4 NCD-SWEET Beamline, ALBA Synchrotron Light Source, 08290 Cerdanyola del Vallès, Spain; esolano@cells.es
5 Facultad de Farmacia de Albacete, Universidad de Castilla-La Mancha, 02008 Albacete, Spain; pilar.ccasares@uclm.es
6 Unidad de Medicina Molecular, Centro Regional de Investigaciones Biomédicas, 02008 Albacete, Spain
7 TA Instruments Waters Chromatography, Tres Cantos, 28760 Madrid, Spain; carlos_gracia@waters.com
8 ID20, ESRF, 71 Avenue des Martyrs, 38000 Grenoble, France; alessandro.longo@esrf.fr
9 Istituto per lo Studio dei Materiali Nanostrutturati (ISMN)-CNR, UOS Palermo, Via Ugo La Malfa, 153, 90146 Palermo, Italy
10 Constellium C-TEC Technology Center, Parc Economique Centr'alp, 725 Rue Aristide Bergès CS10027, 38341 Voreppe, France; georges.salloum-abou-jaoude@constellium.com
11 Experimental Therapeutics Unit, Hospital Clínico San Carlos, IdISSC and CIBERONC, 28040 Madrid, Spain; alberto.ocana@salud.madrid.org
12 Unidad de Investigación del Complejo Hospitalario Universitario de Albacete, Oncología Traslacional, 02008 Albacete, Spain
13 Departamento de Física Aplicada, CINBIO, Universidade de Vigo, Campus Lagoas-Marcosende, 36310 Vigo, Spain; mmpineiro@uvigo.es
14 Netherlands Organisation for Scientific Research (NWO), DUBBLE@ESRF BP CS40220, 38043 Grenoble, France
* Correspondence: cahermida@uvigo.es (C.H.-M.); daniel.hermida@uvigo.es (D.H.-M.)

Table of Contents

Table S1. Correspondence between the abbreviated name and the composition

Figure S1. ^{1}H NMR spectrum (500 MHz, 298 K, $CDCl_3$) of Rac-PLA

Figure S2. ^{1}H NMR spectrum (500 MHz, 298 K, $CDCl_3$) of HC-PLA

Figure S3. ^{1}H NMR spectrum (500 MHz, 298 K, $CDCl_3$) of SC-PLA

Figure S4. ^{1}H NMR deconvoluted spectrum (500 MHz, 298 K, $CDCl_3$) of the homodecoupled CH resonance of Rac-PLA

Figure S5. ^{1}H NMR deconvoluted spectrum (500 MHz, 298 K, $CDCl_3$) of the homodecoupled CH resonance of HC-PLA

Figure S6. 1H NMR deconvoluted spectrum (500 MHz, 298 K, CDCl3) of the homodecoupled CH resonance of SC-PLA

Table S2. DLS analysis of drug-loaded nanoparticles

Table S3. DSC results of the PLA nanoparticles

Figure S7. SEM images of PLA-NP

Figure S8. DSC thermograms of the heating ramp to 65°C of NP-PLA (A) and of the isothermal at 65°C of NP-PLA/DOX

Figure S9. WAXS (A) and SAXS (B) of the gelatin hydrogel

Figure S10. SAXS fitting

Figure S11. DSC thermograms of the Gelatin and NP-PLA bionanocomposites during the heating step from room temperature (A) and the isothermal step at 65°C (B)

Figure S12. Cryo-SEM images of GE with (A-C) and without (B-D) NP-PLA at x2000 (A-B) and x5000 (C-D)

Figure S13. Histograms of the size cell (A) and the size cell above a certain size (B) of the x2000 magnification images for both GE without (w/o NP) and with PLA NPs (w NP). Note that the left side of the bimodal distribution for GE with NP (w NP) the is missing because of the spatial resolution limit of 0.1 μm

Name	Gelatin	PLA	Bulk Polymer/Nanoparticle	Drug
Rac-PLA	**-**	**Racemic**	**Bulk**	**-**
HC-PLA	**-**	**Homopolymer**	**Bulk**	**-**
SC-PLA	**-**	**Stereoblock copolymer**	**Bulk**	**-**
GE	**Yes**	**-**	**-**	**-**
GE/DOX	**Yes**	**-**	**-**	**Doxorubicin**
NP-Rac-PLA	**-**	**Racemic**	**Nanoparticle**	**-**
NP-HC-PLA	**-**	**Homopolymer**	**Nanoparticle**	**-**
NP-SC-PLA	**-**	**Stereoblock copolymer**	**Nanoparticle**	**-**
NP-Rac-PLA/DOX	**-**	**Racemic**	**Nanoparticle**	**Doxorubicin**
NP-HC-PLA/DOX	**-**	**Homopolymer**	**Nanoparticle**	**Doxorubicin**

NP-SC-PLA/DOX	**-**	**Stereoblock copolymer**	**Nanoparticle**	**Doxorubicin**
NP-Rac-PLA/DAS	**-**	**Racemic**	**Nanoparticle**	**Dasatinib**
NP-HC-PLA/DAS	**-**	**Homopolymer**	**Nanoparticle**	**Dasatinib**
NP-SC-PLA/DAS	**-**	**Stereoblock copolymer**	**Nanoparticle**	**Dasatinib**
GE/NP-Rac-PLA/DOX	**Yes**	**Racemic**	**Nanoparticle**	**Doxorubicin**
GE/NP-HC-PLA/DOX	**Yes**	**Homopolymer**	**Nanoparticle**	**Doxorubicin**
GE/NP-SC-PLA/DOX	**Yes**	**Stereoblock copolymer**	**Nanoparticle**	**Doxorubicin**

Table S1. Correspondence between the abbreviated name and the composition

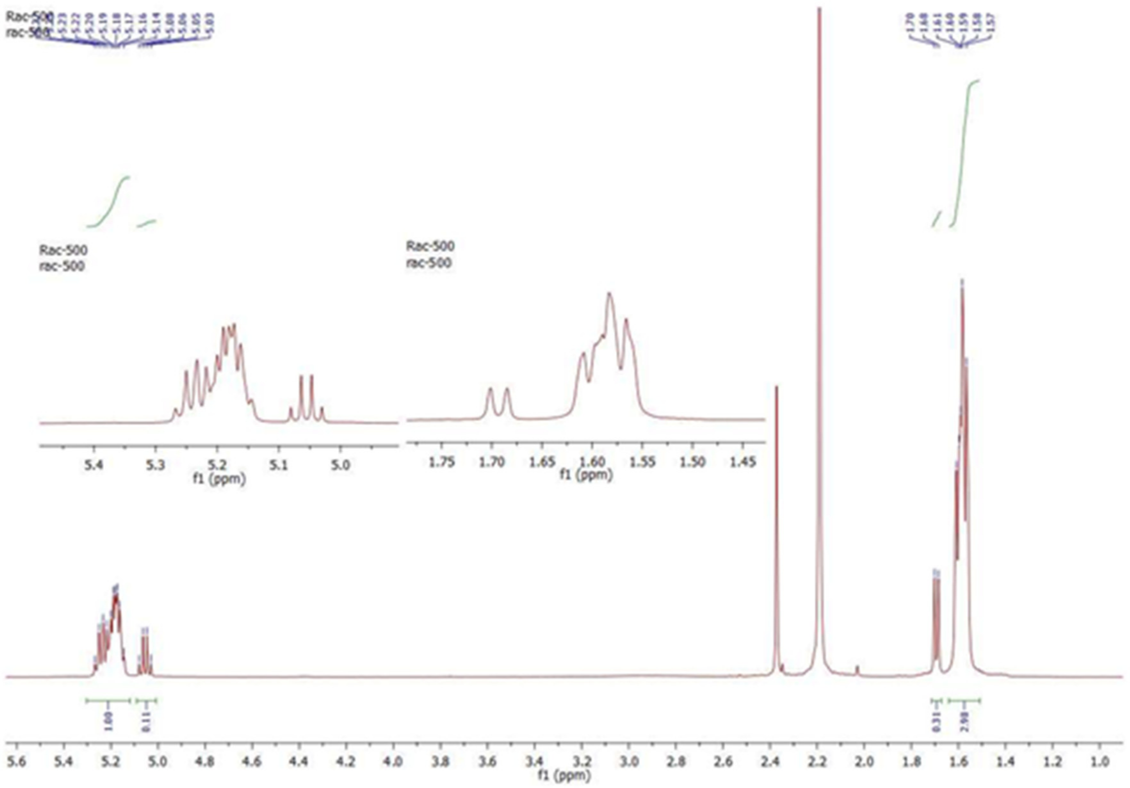

Figure S1. **¹H NMR spectrum (500 MHz, 298 K, $CDCl_3$) of Rac-PLA**

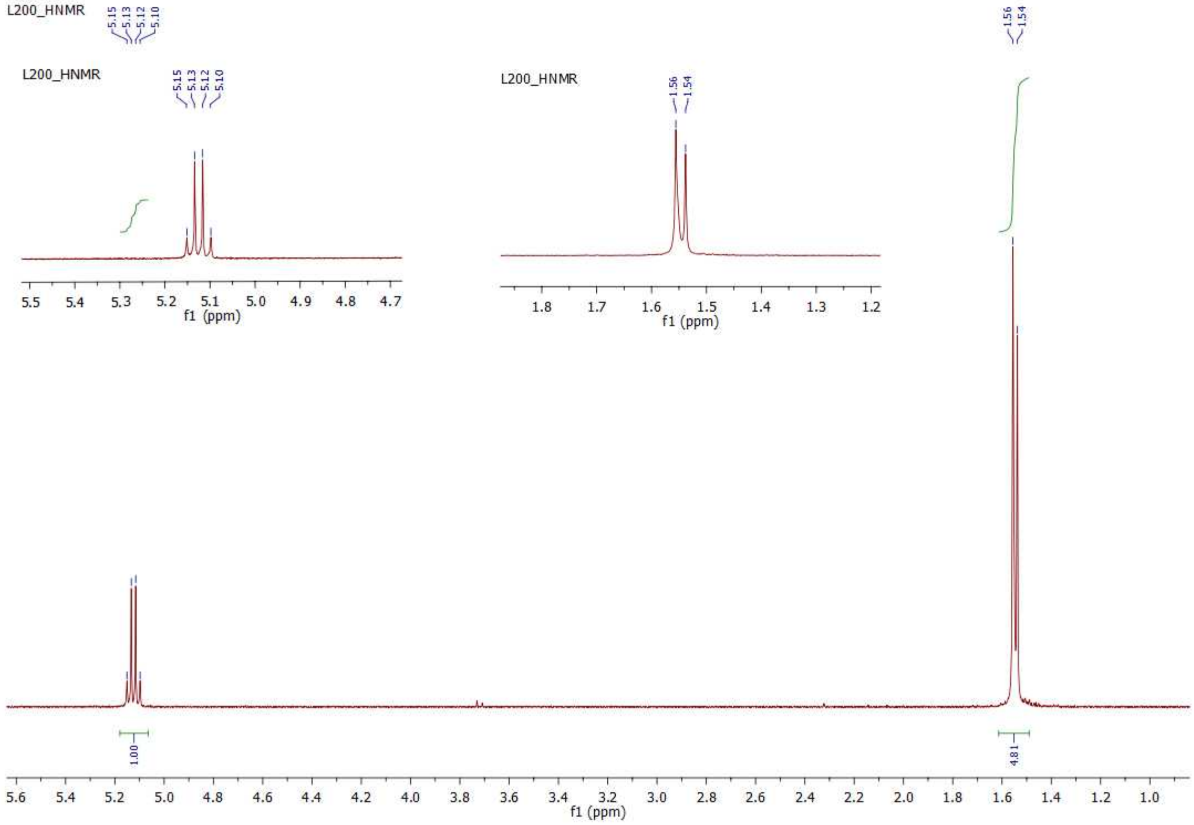

Figure S2. **¹H NMR spectrum (500 MHz, 298 K, $CDCl_3$) of HC-PLA**

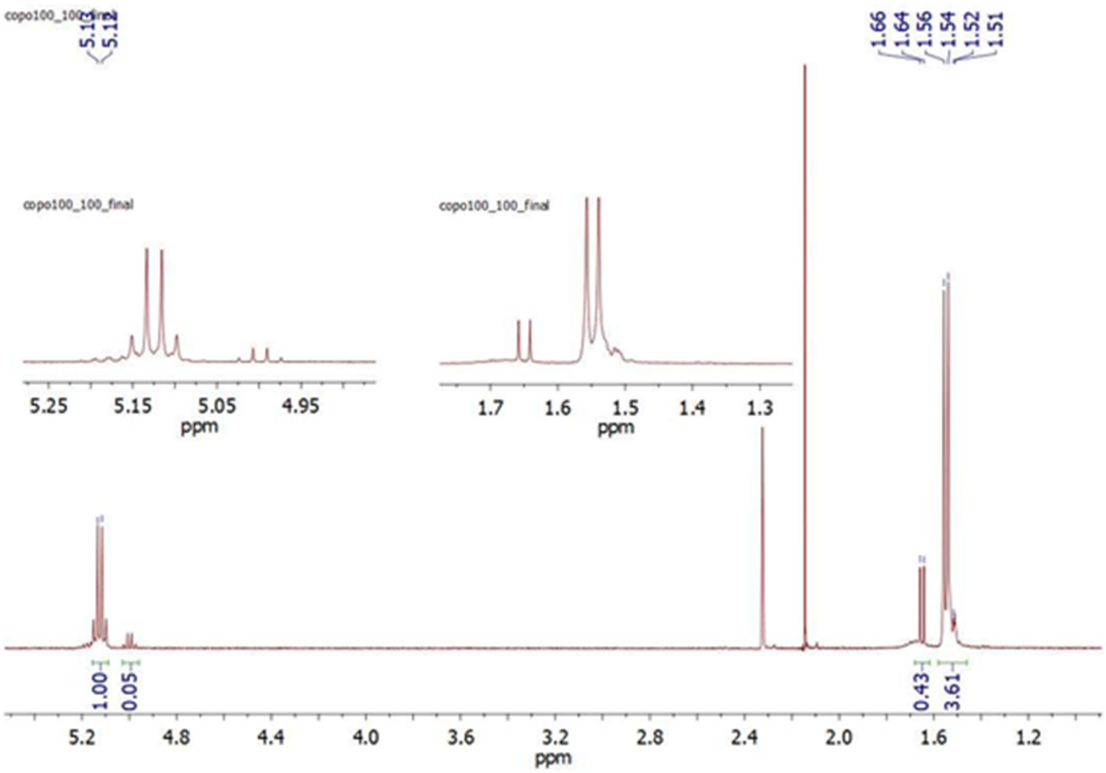

Figure S3. ^{1}H **NMR spectrum (500 MHz, 298 K,** $CDCl_3$**) of SC-PLA**

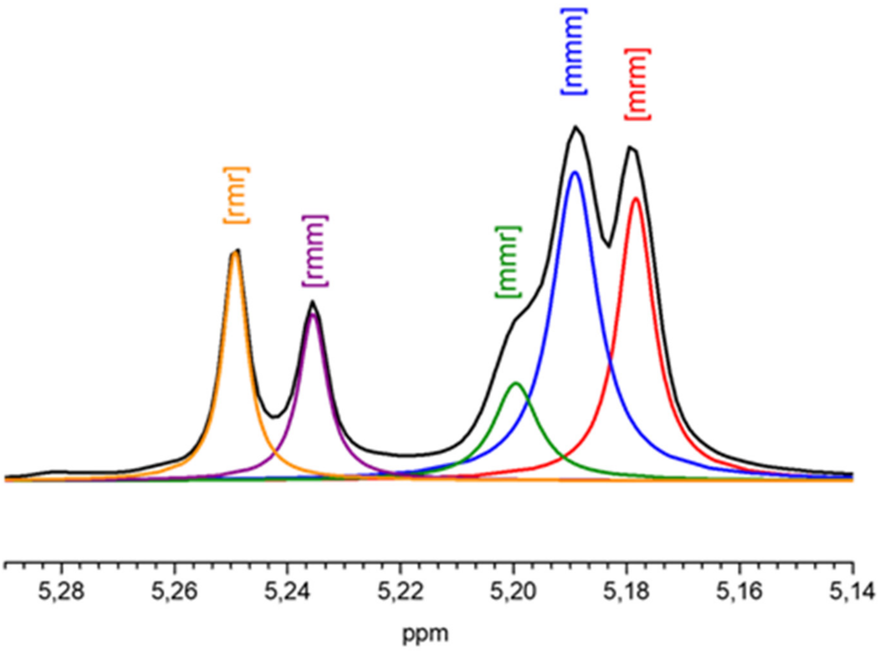

Figure S4. ^{1}H **NMR deconvoluted spectrum (500 MHz, 298 K,** $CDCl_3$**) of the homodecoupled CH resonance of Rac-PLA. Reproduced with permission from Moya-Lopez C., Polymers 14, 232, published by MDPI, 2022.**

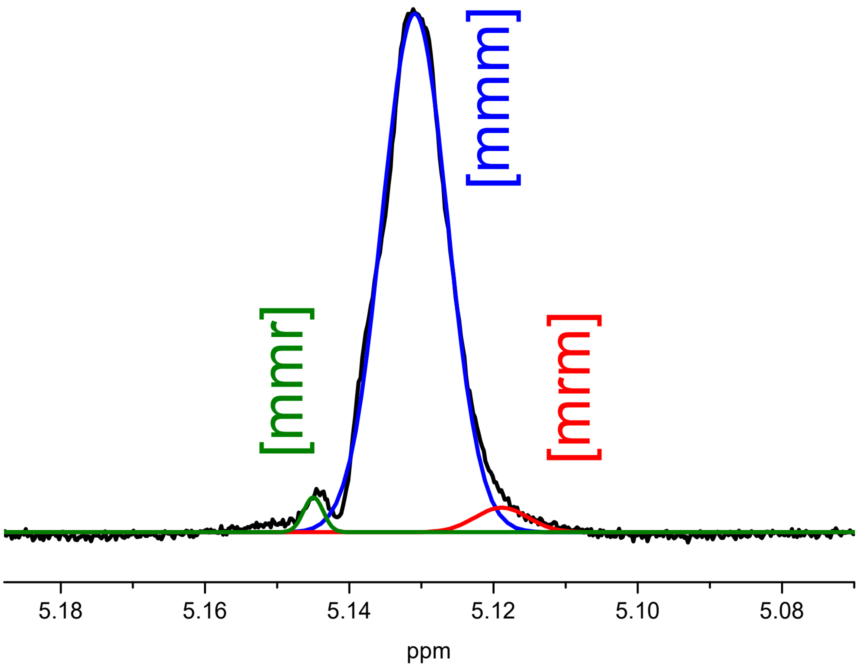

Figure S5. **^{1}H NMR deconvoluted spectrum (500 MHz, 298 K, $CDCl_3$) of the homodecoupled CH resonance of HC-PLA**

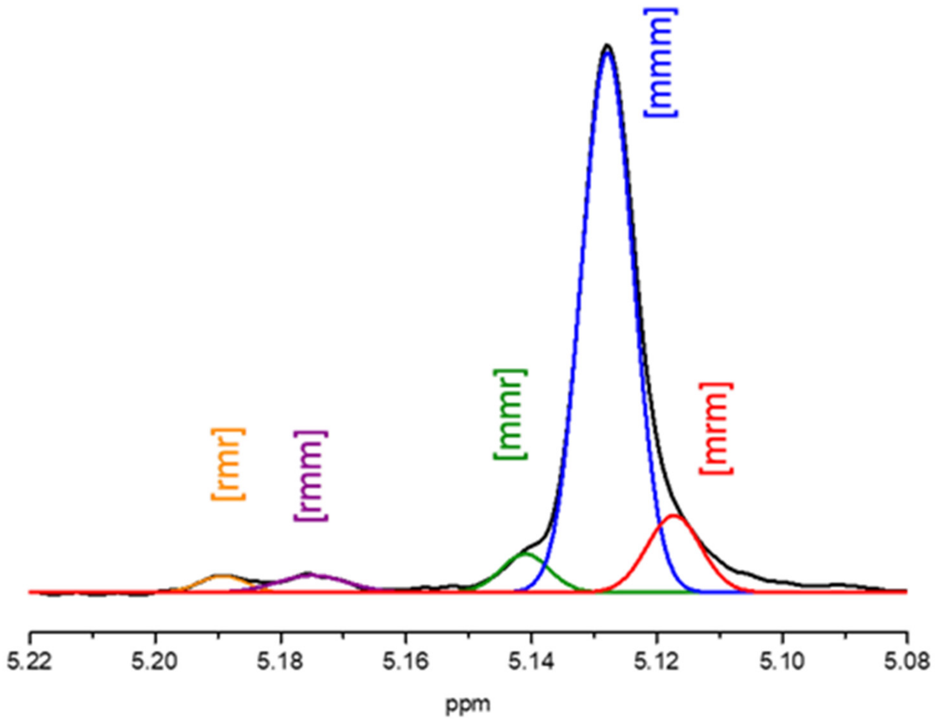

Figure S6. **^{1}H NMR deconvoluted spectrum (500 MHz, 298 K, $CDCl_3$) of the homodecoupled CH resonance of SC-PLA**

Particle	Average size (nm)	PDI	EE [%]
Rac + DOX	286.35	0.17	0.3
HC + DOX	214.8	0.18	0.24
SC + DOX	270.0	0.17	0.18
Rac + DAS	237.6	0.16	1.03
HC + DAS	282.85	0.15	2.8
SC + DAS	275.55	0.17	0.23

Table S2. DLS analysis of drug loaded nanoparticles

Name	T_g (°C)	ΔH_c(J/g) (T_c= 65°C)	T_m (°C)	ΔH_m(J/g)	X_m (%)
NP-Rac-PLA	40.63	-	-	-	
NP-HC-PLA	40.84	46.102	165.27	22.13	23.61
NP-SC-PLA	39.99	96.5	197.35	45.19	31.8

Table S3. DSC results of the PLA nanoparticles

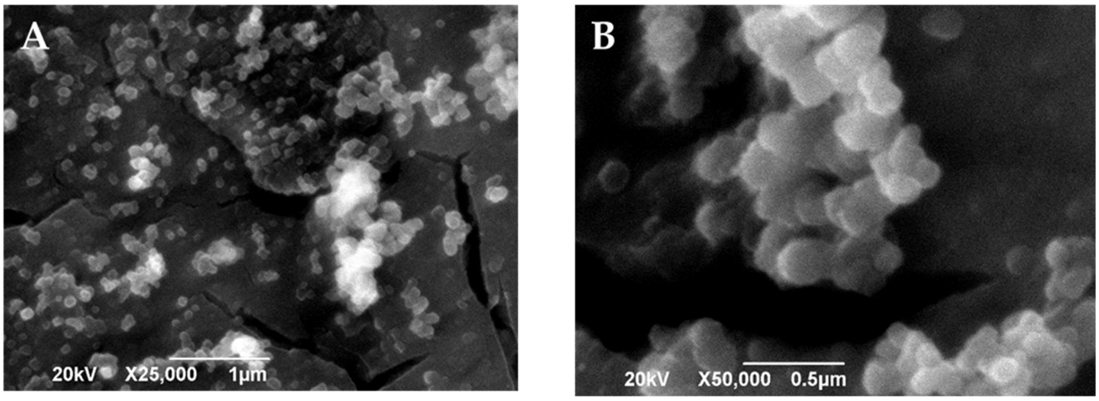

Figure S7. SEM images of PLA-NP at x25 000 (A) and x50.000 (B) magnification

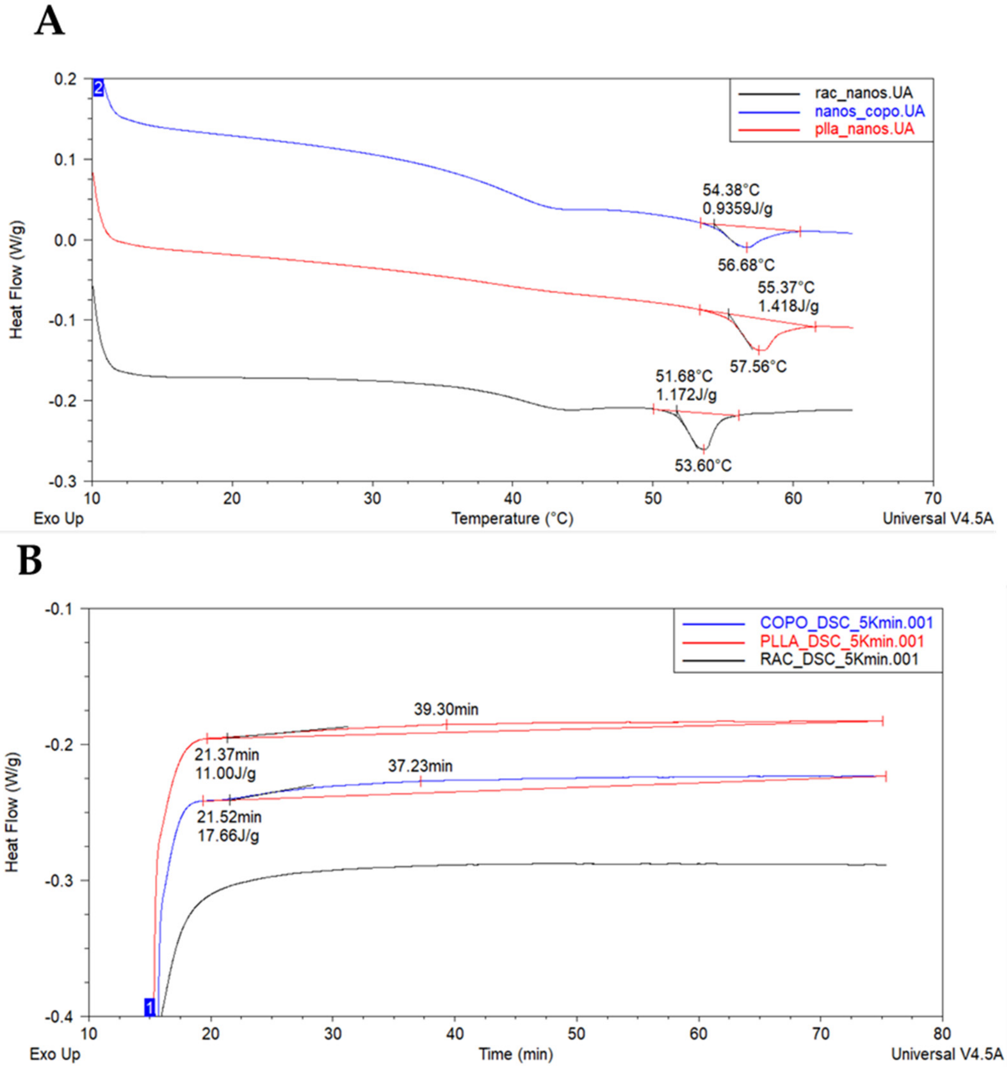

Figure S8. DSC thermograms of the heating ramp to 65°C of NP-PLA (A) and of the isothermal at 65°C of NP-PLA/DOX (B)

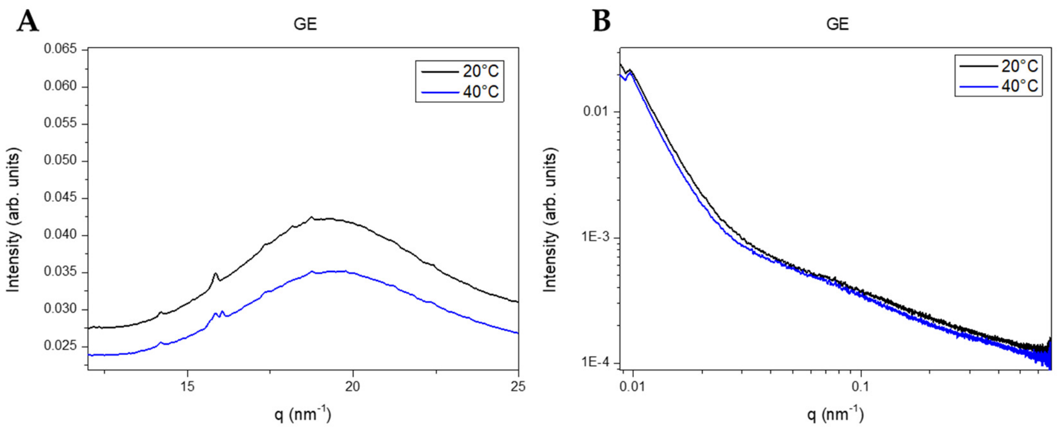

Figure S9. WAXS (A) and SAXS (B) of the gelatin hydrogel

	Sample		
Parameter	Ge+ NP 25°C	Ge+ NP 65°C_s	Ge+ NP 65°C_e
rsp	1.0000	0.33696	0.65245
csi	6.2430	1.2424	0.80573
df	1.0115	2.5157	2.9562

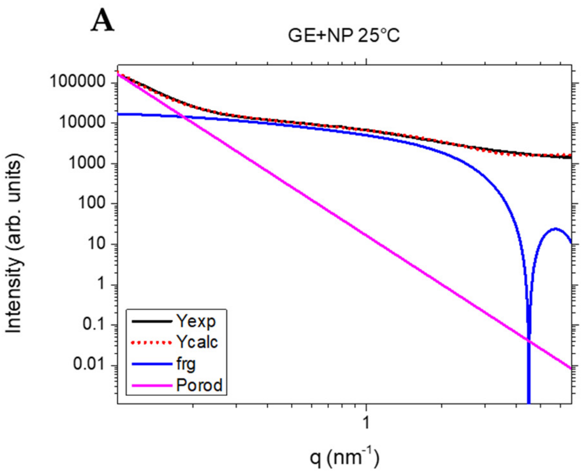

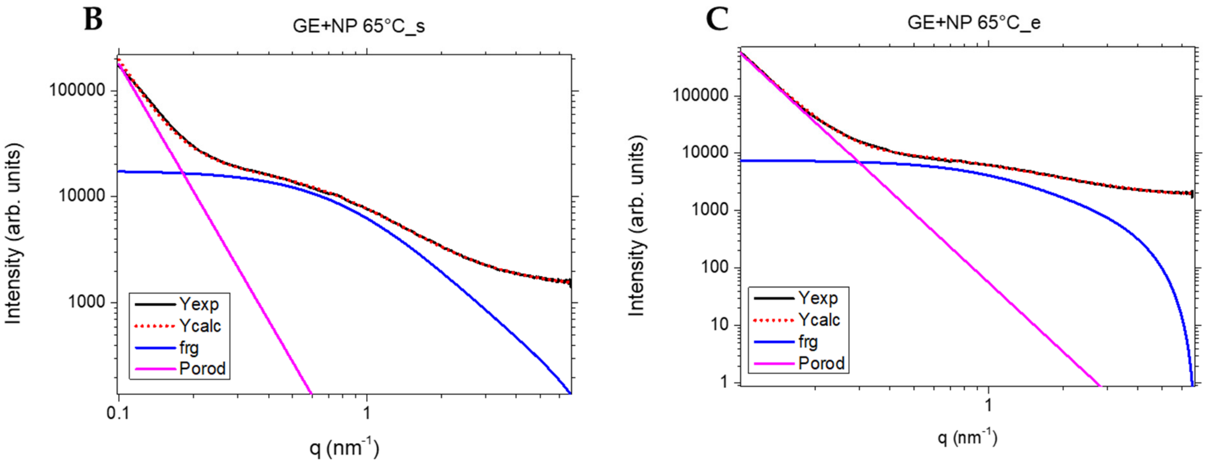

Figure S10. Table S4.SAXS fitting parameters and plots of GE/NP-HC-PLA at 25°C (A), 65°C (B) and after 30 min at 65°(C).

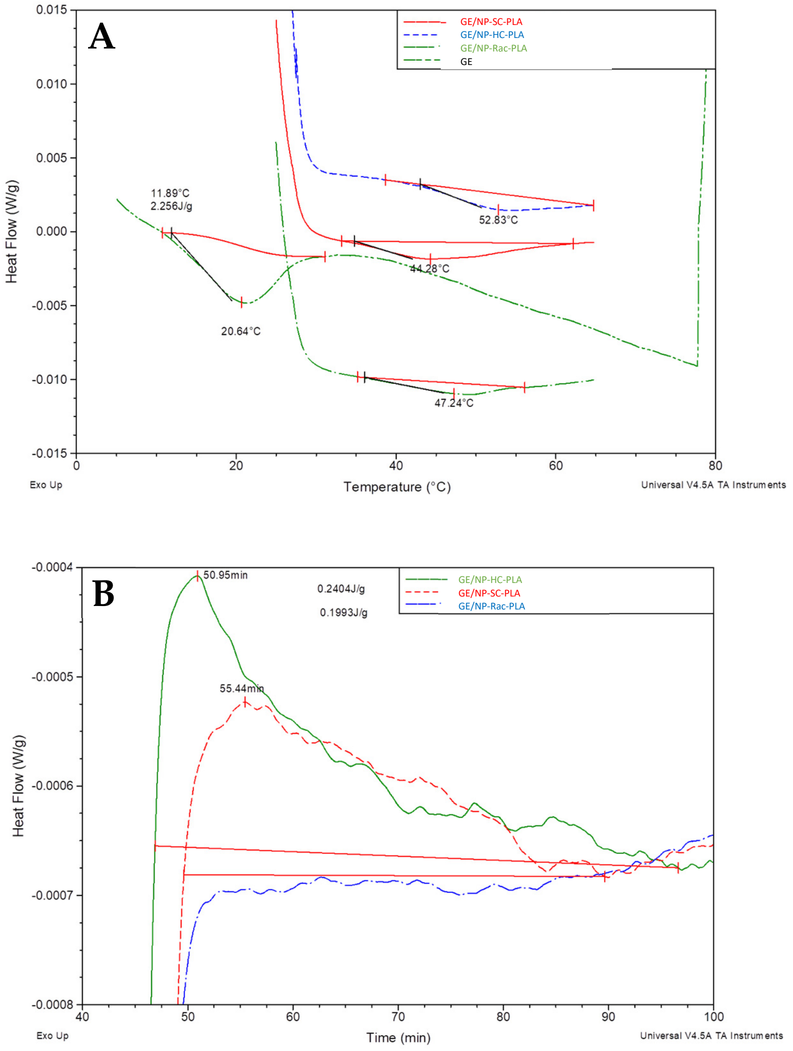

Figure S11. DSC thermograms of the Gelatin and NP-PLA bionanocomposites during the heating step from room temperature (A) and the isothermal step at 65°C (B).

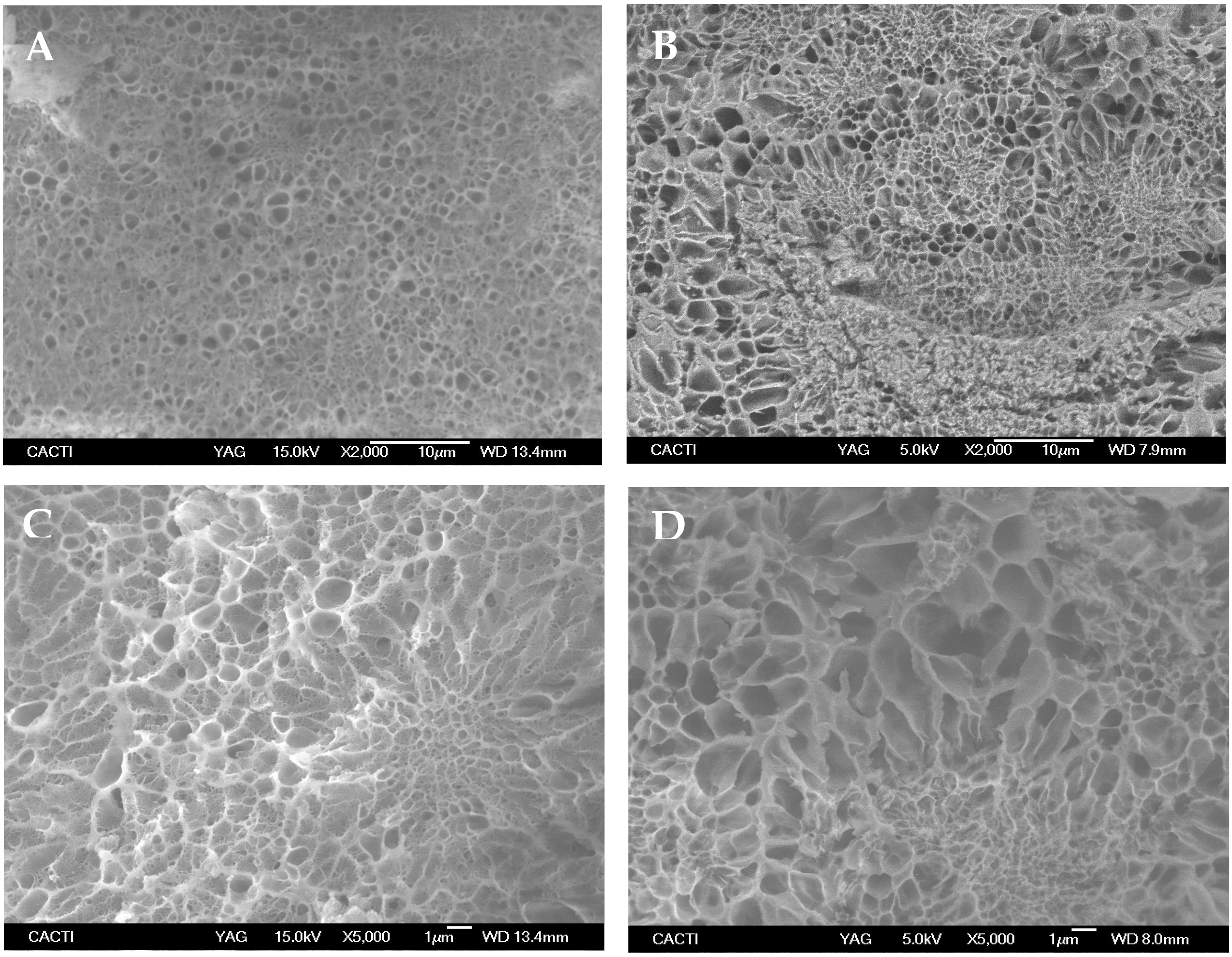

Figure S12. Cryo-SEM images of GE with (A-C) and without (B-D) NP-PLA at x2000 (A-B) and x5000 (C-D) magnification.

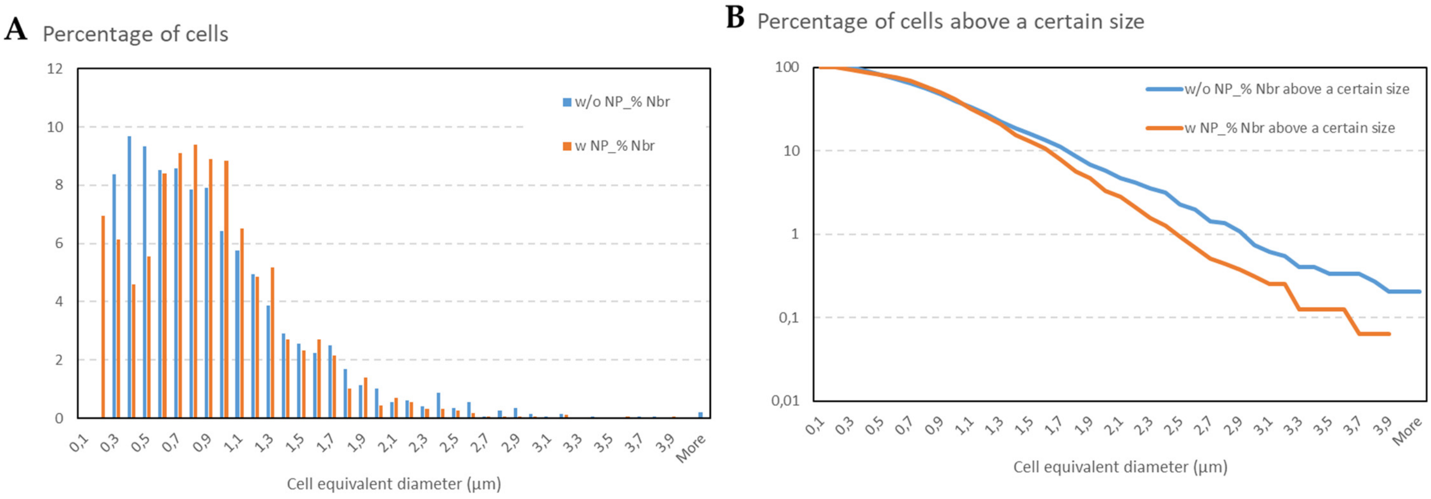

Figure S13. Histograms of the size cell (A) and the size cell above a certain size (B) of the x2000 magnification micrographs for both GE without (w/o NP) and with PLA NPs (w

NP). Note that the left side of the bimodal distribution for GE with NP (w NP) is missing because of the spatial resolution limit of 0.1 μm
